# Perforated Appendicitis Presenting as Small Bowel Obstruction in an Infant

**Published:** 2011-11-27

**Authors:** Bilal Mirza

**Affiliations:** Department of Paediatric Surgery, The Children's Hospital and the Institute of Child Health Lahore, Pakistan

**Dear Sir**

Acute appendicitis is one of the frequently seen emergencies in the pediatric hospitals. Nevertheless, its diagnosis and management is often challenging in infants and neonates. The diagnosis is frequently made at operation performed for the complications [1, 2, 3]. We encountered a similar situation in a 2-month-old infant in whom the perforated appendicitis presented with small bowel obstruction.

A 2-month-old male infant presented to the surgical emergency with 4-day history of bilious vomiting, abdominal distension, constipation, irritability, and reluctance to feed. Two days prior to presentation he developed moderate intensity fever without rigors. There was no history of diarrhea or respiratory tract infection. General physical examination showed temperature 100F, respiratory rate 30 breaths/min, and pulse of 100 beats/min. On examination, abdomen was distended and mildly tender in the right lower quadrant. No mass or viscera were palpable. A digital rectal examination showed mucous in the rectum.

The laboratory investigations (complete blood counts, serum electrolytes, and urinalysis) were normal. Abdominal radiograph showed multiple air fluid levels (Fig. 1). Ultrasound of the abdomen showed adynamic fluid filled bowel loops especially in the lower abdomen. At operation, multiple loops of small bowel were adherent in the right iliac fossa. On adhesionolysis, a perforated appendix without pus was found. Appendectomy was performed. The post operative recovery was uneventful except for wound infection. 

**Figure:**
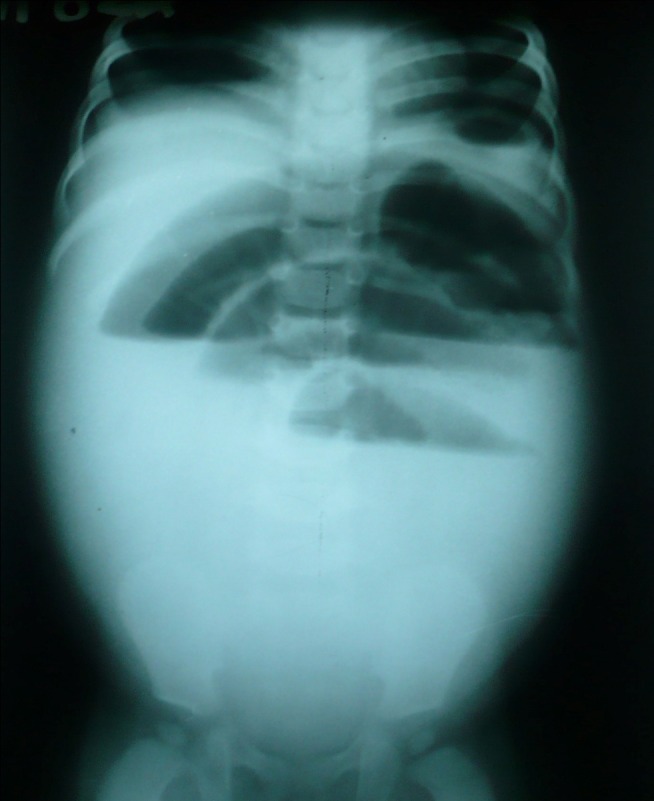
Figure 1: Abdominal radiograph showing air fluid levels

It is estimated that about 8% of children who present with abdominal pain are ultimately diagnosed with acute appendicitis. It is very uncommon in infants and neonates. Only 2% patients treated for acute appendicitis are below 2 years of age. The rate of perforation of appendix is 30% in adults as compared to 98% in infants and neonates [1, 2, 3].

The common clinical features in infants and toddlers who present with appendicular perforation are abdominal pain, vomiting, fever, lethargy, and reluctance to feed. These are nonspecific features but a high index of suspicion may lead to the accurate diagnosis. In case of delayed diagnosis, the appendix may perforate and result in localized or generalized peritonitis, appendicular abscess and appendicular mass. Occasionally intestinal loops may adhere to the inflamed and perforated appendix limiting the spread of peritonitis [2, 3].

## Footnotes

**Source of Support:** Nil

**Conflict of Interest:** None declared
